# Identification of a novel prognostic DNA methylation signature for lung adenocarcinoma based on consensus clustering method

**DOI:** 10.1002/cam4.3343

**Published:** 2020-08-28

**Authors:** Qidong Cai, Boxue He, Hui Xie, Pengfei Zhang, Xiong Peng, Yuqian Zhang, Zhenyu Zhao, Xiang Wang

**Affiliations:** ^1^ Department of Thoracic Surgery The Second Xiangya Hospital Central South University Changsha Hunan China; ^2^ Hunan Key Laboratory of Early Diagnosis and Precision Therapy Department of Thoracic Surgery The Second Xiangya Hospital Central South University Changsha Hunan China

**Keywords:** biomarker, consensus clustering, DNA methylation, lung adenocarcinoma

## Abstract

Abnormal DNA methylation persists throughout carcinogenesis and cancer development. Hence, gene promoter methylation may act as a prognostic tool and provide new potential therapeutic targets for patients with lung adenocarcinoma (LUAD). In this study, to explore prognostic methylation signature, data regarding DNA methylation and RNA‐seq, and clinical data of patients with LUAD from the Cancer Genome Atlas database (TCGA) were downloaded. After data preprocessing, the methylation data were divided into training (N = 405) and test sets (N = 62). Then, patients in the training set were assigned to five subgroups based on their different methylation levels using the consensus clustering method. We comprehensively analyzed the survival information, methylation levels, and clinical variables, including American Joint Committee on Cancer (AJCC) stage, tumor‐node‐metastasis (TNM) staging, age, smoking history, and gender of these five groups. Subsequently, we identified a 16‐CpG prognostic signature and constructed a prognostic model, which was verified in the test set. Further analyses showed stable prognostic performance in the stratified cohorts. In conclusion, the new predictive DNA methylation signature proposed in this study may be used as an independent biomarker to assess the overall survival of LUAD patients and provide bioinformatics information for development of targeted therapy.

## Graphical abstract

In this study, based on consensus clustering method, we identified and validated a robust prognostic DNA methylation signature.

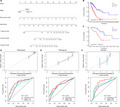

## 1 INTRODUCTION

Lung cancer is the most common type of cancer and the leading cause of cancer‐related deaths worldwide.^1^ Non‐small cell lung cancer (NSCLC) accounts for approximately 85% cases of lung cancer, and lung adenocarcinoma (LUAD) is the major type of NSCLC. Although various personalized treatment strategies have been developed for treating LUAD, the survival outcome of LUAD is still poor because of tumor invasion and metastasis, with an average 5‐year survival rate of 15%.^2^, ^3^, ^4^ Considering the serious challenges associated with LUAD, the identification of prognostic signatures is of considerable importance for improving LUAD diagnosis, prognosis, and treatment.

DNA methylation is a process via which methyl groups are added to the DNA, mostly on CpG motifs. Methylation at the transcriptional start site (TSS) often suppresses gene expression. Abnormal methylation of CpG sites and subsequent inactivation of pivotal tumor suppressor genes have been widely recognized as important mechanisms of tumorigenesis.^5^ In addition, as an epigenetic modification, DNA methylation is reversible, which renders identification of therapeutic targets promising. Consequently, aberrant DNA methylation patterns can be utilized as ideal biomarkers for LUAD diagnosis, prognosis, and drug sensitivity.^6^, ^7^ Studies have identified aberrant methylation of multiple genes in LUAD. Furthermore, a previous study demonstrated that nine methylation‐driven genes can be used as prognostic biomarkers for patients with LUAD.^8^ Wang et al identified a 16‐CpG‐based model to estimate the probability of mortality for patients with LUAD.^9^ Despite these observations, more biomarkers for LUAD are required for risk stratification and identification of novel therapeutic targets in patients with LUAD.

LUAD is a highly heterogeneous cancer.^10^ Tumor heterogeneity at the population level, a hallmark of tumors, confounds the diagnosis of LUAD and challenges subsequent treatment owing to the presence of diverse genetic mutations and epigenetic modifications. As one of the important epigenetic factors contributing to tumor heterogeneity, the extent of DNA methylation partially reflects the heterogeneity level within a tumor.^11^, ^12^ Therefore, dividing LUAD samples into different clusters according to their methylation status and analyzing the key CpGs contributing to heterogeneity and lethal outcome of LUAD appear to be a feasible approach. Traditional clustering methods have several drawbacks, which may not yield satisfactory clustering results. For example, hierarchical clustering is unable to provide “objective” criterion for determining the number of clusters and cluster boundaries. Although iterative descent clustering methods, such as k‐means clustering, eliminate some of the defects of hierarchical clustering, the number of clusters still have to be predefined; in addition, a uniform standard for comparing the clustering results from different clustering number is lacking, along with dearth of methods for validating the reliability and rationality of the above clustering approaches.^13^ Consensus clustering method, a resampling‐based method that can integrate data from a patient cohort and divide patients into apparently stable clusters for a range of k, where k is a predefined number of clusters, can overcome these shortcomings and has been introduced for this purpose.^13^ Owing to its robust results and good visualization of cluster composition and number, this methodology is now widely used in genomic studies.^14^, ^15^, ^16^

The Cancer Genome Atlas (TCGA) has cataloged the transcriptomic and epigenetic profiles of 33 types of human cancer.^17^ This allows integration of data from multiple perspectives, including the transcriptome and methylome, and clinical information, to assess the potential of CpGs for predicting the outcomes of LUAD. In the present study, we aimed to perform a comprehensive analysis and developed a 16‐CpG methylation signature to predict the prognosis and assist clinicians in managing patients with LUAD. Considering the complementary values of molecular and clinicopathological features, we built a prognostic nomogram that integrated the methylation signature and American Joint Committee on Cancer (AJCC) stage. This nomogram enables improved estimation of LUAD prognosis and these identified methylation biomarkers could lay the groundwork for personalized precision cancer medicine.

## 2 METHODS

### 2.1 Data collection and preprocessing

DNA methylation datasets of 503 LUAD samples from the Illumina Human Methylation 450 platform (450K array) and 150 LUAD samples from the Illumina Human Methylation 27 platform (27K array) were downloaded from the UCSC Xena database (https://xenabrowser.net/datapages/).^18^ Methylation at each CpG site is described by the β value, which is the ratio between the intensities of the methylated probe and total probe. RNA‐seq results in fragments per kilobase of transcript per million mapped reads (FPKM) format from 493 primary LUAD samples were downloaded from TCGA data portal (https://portal.gdc.cancer.gov/). The clinical information for these patients is shown in Table S1. The study was conducted strictly in compliance with the publication guidelines approved by TCGA.

The clinical data were preprocessed by removing samples with missing survival state and overall survival (OS) information. To remove samples pertaining to non‐cancer‐related death, patients with OS less than 30 days were not included in the study. Regarding methylation data, CpG sites that were absent in >70% of the samples were deleted. The sites of single‐nucleotide polymorphisms or those located in sex chromosomes were also excluded. As methylation of CpG sites in promoter regions significantly affects gene expression, we defined the gene's promoter region as the genomic interval 2000 bp upstream and 500 bp downstream of TSS and specifically investigated CpGs in this region.^19^ The remaining sites, the data of which were not available (NA), were imputed using the k‐nearest neighbors (KNN) imputation method.

The data of 450K and 27K arrays were designated as the training set and the test set, respectively. Methylation data from two sets were corrected for batch effects using the ComBat method of the sva software package.^20^

### 2.2 Screening of survival‐related CpGs

Cox regression analyses were conducted to identify CpG sites significantly related to OS of LUAD. First, we performed univariate Cox regression analysis for preliminary screening of survival‐related CpG sites. Then, the CpGs identified using univariate Cox regression were used for multivariate Cox regression together with clinical parameters, including age, gender, smoking history, tumor (T) category, node (N) category, metastasis (M) category, and AJCC stage, to further select prognostic CpG sites independent of these clinical data. CpG sites that were significant in both univariate and multivariate Cox regression analyses were selected for subsequent study.

### 2.3 Determination of molecular subtypes based on methylation levels using consensus clustering

Consensus clustering was performed using the ConsensusClusterPlus software package^21^ to assign LUAD samples into different clusters according to their independent prognostic CpG sites’ methylation levels. In the present study, the algorithm subsampled 80% samples and 80% CpGs from a data matrix. Subsequently, each subsample was partitioned into k groups using k‐means. The sampling process was repeated 50 times. Pairwise consensus values, defined as “the proportion of clustering runs in which two items are clustered together”, were calculated for each k and stored in a consensus matrix to represent and quantify the agreement among the clustering runs over the resampled datasets. Finally, for each k, an agglomerative hierarchical consensus clustering using a distance of 1‐consensus value was completed and pruned to k groups. In summary, this algorithm determined “consensus clusters” by evaluating the stability of clustering outputs of the k‐means clustering analysis in randomly subsampled datasets.^13^

### 2.4 Analysis of survival and clinical features

OS differences between different clusters were assessed using the Kaplan‐Meier method and compared using the log‐rank test. Percentage plots were plotted to illustrate the intercluster differences of clinical features among these groups. The Chi‐square test was performed to compare differences between clusters. *P* value < .05 was considered statistically significant for the log‐rank test or Chi‐square test.

### 2.5 Identification of cluster‐specific CpGs

We performed pairwise Wilcoxon comparisons to identify cluster‐specific CpGs that contribute to the differences among clusters. The methylation level of these CpGs in every two groups was also compared using the Kruskal‐Wallis test. The *P* values from pairwise comparisons were adjusted using Benjamini‐Hochberg correction via the *p.adjust* function of the R software.^22^ The criteria for screening out cluster‐specific methylation CpG sites were false discovery rate (FDR) < 0.05 and |Fold Change| > 1.5.

### 2.6 Construction and evaluation of prognostic model

A stepwise model selection by the Akaike information criterion (AIC) was conducted to select the final list of CpG sites from cluster 5. Finally, the risk score of the prognostic model was calculated as follows: $\text{Risk score}=\sum {\text{coef}}_{i}\times \beta_{i}$, in which coef_i_ represents the regression coefficient of CpG site *i*, and *β*_i_ is the *β* value of the corresponding site.

To evaluate the efficacy of the risk model, we performed survival analysis and receiver operating characteristic (ROC) analysis on the training and test sets, respectively. In addition, stratified analysis was performed on the 450 K data. As major driver mutations in LUAD, *KRAS* and *EGFR* mutations are critically implicated in the pathogenesis of LUAD, and these two genes have emerged as important therapeutic targets for the treatment of LUAD.^23^, ^24^ Hence, we extracted the somatic mutation data of *EGFR* and *KRAS*, processed using the VarScan software, from TCGA database and used them for stratified analysis together with clinicopathological parameters, including AJCC stage, lymph node metastasis (LNM) status, distant metastasis (DM) status, and diagnostic age. We also analyzed the relationships between risk score model and important risk factors for patients with LUAD using the Wilcoxon test.

### 2.7 Nomogram development and evaluation of predictive performance

To improve the prediction accuracy of the risk score model and provide a quantitative method for clinicians to predict the OS of patients with LUAD, we generated a nomogram after integrating the prognostic model and AJCC stage. The predictive performance of the nomogram was assessed via discrimination and calibration. The discrimination of the nomogram was determined using the ROC curve and the concordance index (C‐index). The C‐index value ranged between 0.5 and 1.0, in which C‐index equal to 0.5 suggests random chance and 1.0 indicates perfect discrimination power. The calibration of the nomogram was determined using the calibration curve, which graphically depicts the agreement between the prediction probabilities of the nomogram and the probabilities observed.

### 2.8 Gene set enrichment analysis (GSEA)

To investigate the mechanisms associated with the high‐risk group, GSEA was performed using the GSEA software (http://www.broadinstitute.org/gsea). Gene expression data of 396 available patients with LUAD in the training group were analyzed by the GSEA software using C2.KEGG and C5.BP MSigDB datasets from the Broad Institute.^25^ The GSEA results with normalized enrichment score and *P* value were obtained and exported to the Enrichment Map application in the Cytoscape (http://cytoscape.org/)^26^ software for further analysis. GSEA output files with the following cut‐off parameters were used to build the enrichment map: *P* < .05, *FDR* *q*‐value < 0.1, and overlap similarity coefficient > 0.5.

### 2.9 Statistical analysis

The Wilcoxon test was applied for pairwise comparison among five groups for cluster‐specific CpG sites and analysis of the relationships between the risk score model and important risk factors. The Kruskal‐Wallis test was used to compare the methylation level of every two groups. Categorical data were compared using the Chi‐square test. The risk score formula was calculated using multivariate Cox hazard model analysis. The median value of risk score in the training set was used as the cut‐off for the high‐risk and low‐risk groups. Kaplan‐Meier and log‐rank analyses were performed to assess prognosis.

All the statistical analyses were performed using R (version 3.6.2; https://www.r‐project.org). All analyses were two‐tailed, and *P* value < .05 was considered statistically significant. Benjamini‐Hochberg FDR adjustment was used to correct multiple comparisons. Survival, Cox regression, and ROC analyses were performed using the survival and survivalROC package. The nomogram was generated using the rms package. Corresponding survival curve, ROC curve, risk plot, and heat map were generated using ggplot2 and pheatmap packages.

## 3 RESULTS

### 3.1 Identification of survival‐related CpG sites

**FIGURE 1:**
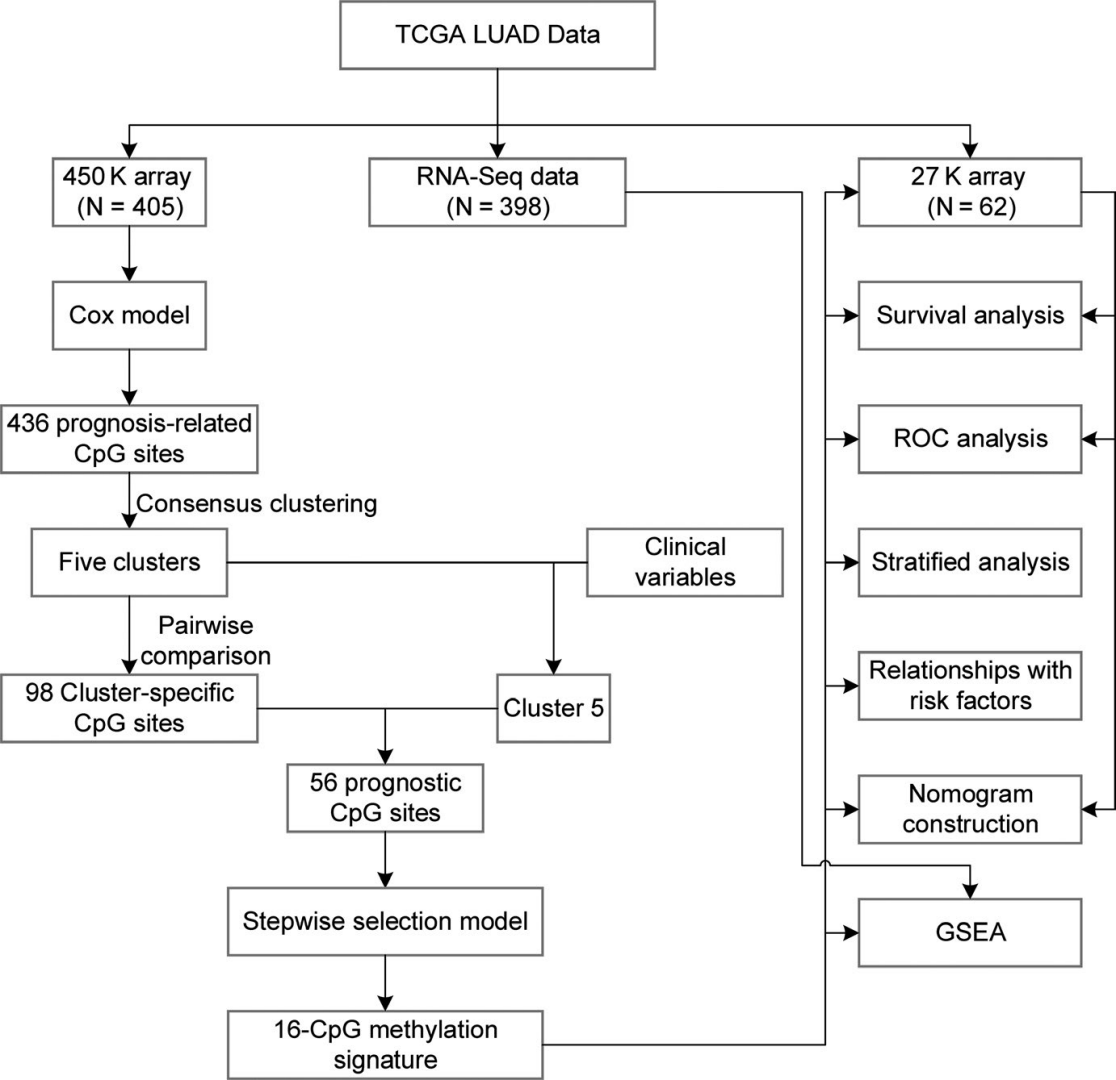
Flowchart of the present study

The flowchart of this study is shown in Figure 1. In total, 405 LUAD samples and 21,220 CpGs from the training set (clinical information in Table S2), as well as 62 LUAD samples and 21,220 CpGs from the test set (clinical information in Table S3) were included in the following analyses. Detailed clinical and pathological information of these patients are shown in Table 1. First, 1044 CpGs (Table S4) were identified as OS‐related CpG sites using univariate Cox regression analysis. Subsequently, multivariate Cox analysis performed on these CpG sites with covariates, including age, gender, smoking history, AJCC stage, and tumor‐node‐metastasis (TNM) staging, identified 436 methylation (Table S5) sites as independent OS‐related CpG sites.

**TABLE 1:** Clinical and pathological parameters of patients involved in the present research

	Training set	Test set	*P* value
n	405	62	
Gender (%)			.775
Female	217 (53.6)	35 (56.5)	
Male	188 (46.4)	27 (43.5)	
Age (%)			.271
<=65	199 (50.4)	26 (41.9)	
>65	196 (49.6)	36 (58.1)	
Smoking history (%)			.889
Nonsmoker	57 (14.5)	8 (12.9)	
Smoker	336 (85.5)	54 (87.1)	
AJCC stage (%)			.578
Stage I	222 (55.5)	33 (55.9)	
Stage II	95 (23.8)	11 (18.6)	
Stage III	64 (16.0)	10 (16.9)	
Stage IV	19 (4.8)	5 (8.5)	
T classification (%)			.351
T1	142 (35.3)	15 (24.2)	
T2	213 (53.0)	39 (62.9)	
T3	31 (7.7)	6 (9.7)	
T4	16 (4.0)	2 (3.2)	
N classification (%)			.003
N0	265 (66.9)	38 (61.3)	
N1	73 (18.4)	14 (22.6)	
N2	57 (14.4)	7 (11.3)	
N3	1 (0.3)	3 (4.8)	
M classification (%)			.864
M0	260 (93.5)	57 (91.9)	
M1	18 (6.5)	5 (8.1)	
OS_event (%)			.118
Alive	309 (76.3)	41 (66.1)	
Dead	96 (23.7)	21 (33.9)	
OS_time (median [min, max])	1.78 [0.09, 19.86]	2.48 [0.10, 10.07]	.031

Abbreviation: OS, overall survival.

### 3.2 Consensus clustering according to molecular subtypes

**FIGURE 2:**
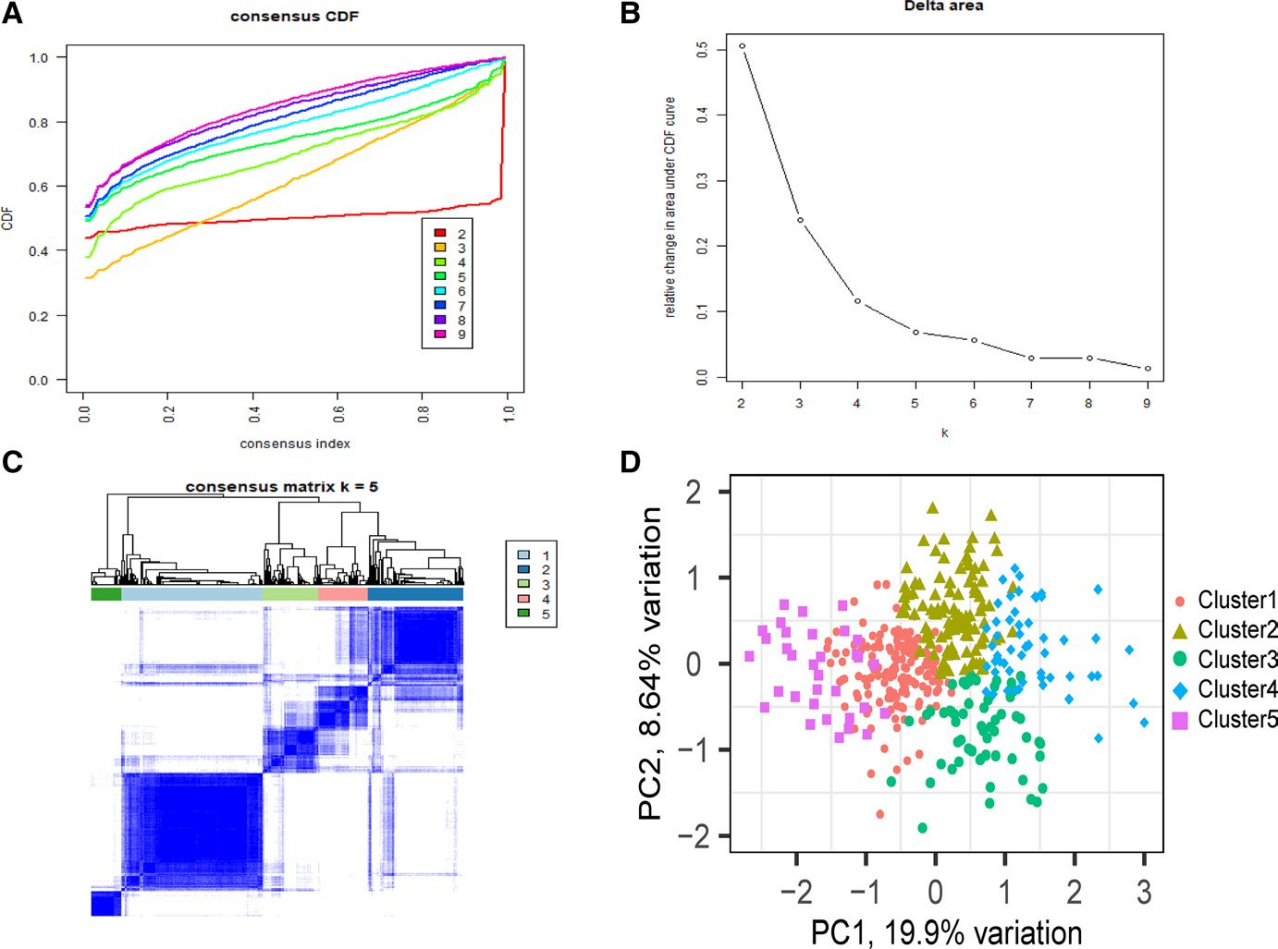
Consensus clustering results based on the methylation of lung adenocarcinoma (LUAD) samples in the training set. A, Empirical cumulative distribution function (CDF) plot displaying consensus distributions for each *k*. B, Delta area plot reflecting the relative changes in the area under the CDF curve. C, Consensus matrix heat map depicting consensus values on a white to blue color scale of each cluster. D, Principal component analysis (PCA) plot

**FIGURE 3:**
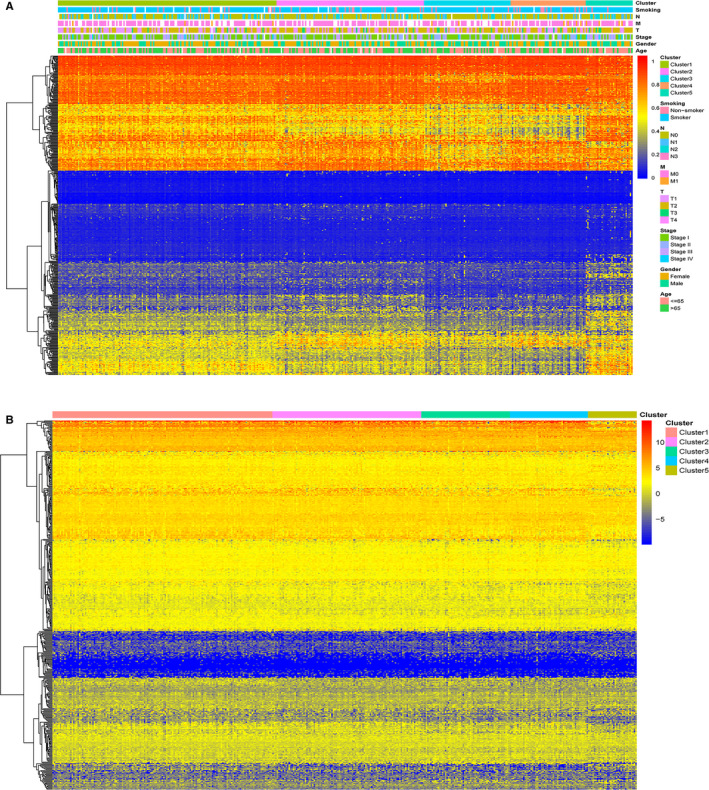
Heat maps showing methylation and corresponding gene expression profile of the 436 CpG sites. A, Heat map illustrating the methylation of each cluster. The blank in the annotation bar refers to not available (NA) clinical traits. B, Heat map displaying the expression profile of the 484 annotated genes. The gene expression of cluster 5 differed slightly from those of other clusters. The expression data of the annotated genes were log2 transformed

Consensus clustering was performed on samples of 450K array according to the methylation profile of the 436 potential prognostic CpG sites. The results of consensus clustering were visualized using an empirical cumulative distribution function (CDF) plot and a delta area plot (Figure 2A,B), in which k represents the number of subgroups. Both plots were plotted to dissect the optimal k value at which the sample distribution was stable. We selected *k* = 5 as the ideal number of clusters. A consensus matrix heat map and a principal component analysis (PCA) plot (Figure 2C,D) further validated the LUAD cohort and divided it into five distinct and nonoverlapping subgroups. A heat map corresponding to the dendrogram in Figure 2C is shown in Figure 3A, which demonstrates the distinct methylation of different groups. The related annotated gene expression heat map is shown in Figure 3B, in which slight differences can be observed between cluster 5 and the other four clusters.

### 3.3 Survival and clinical feature analysis of the five clusters

**FIGURE 4:**
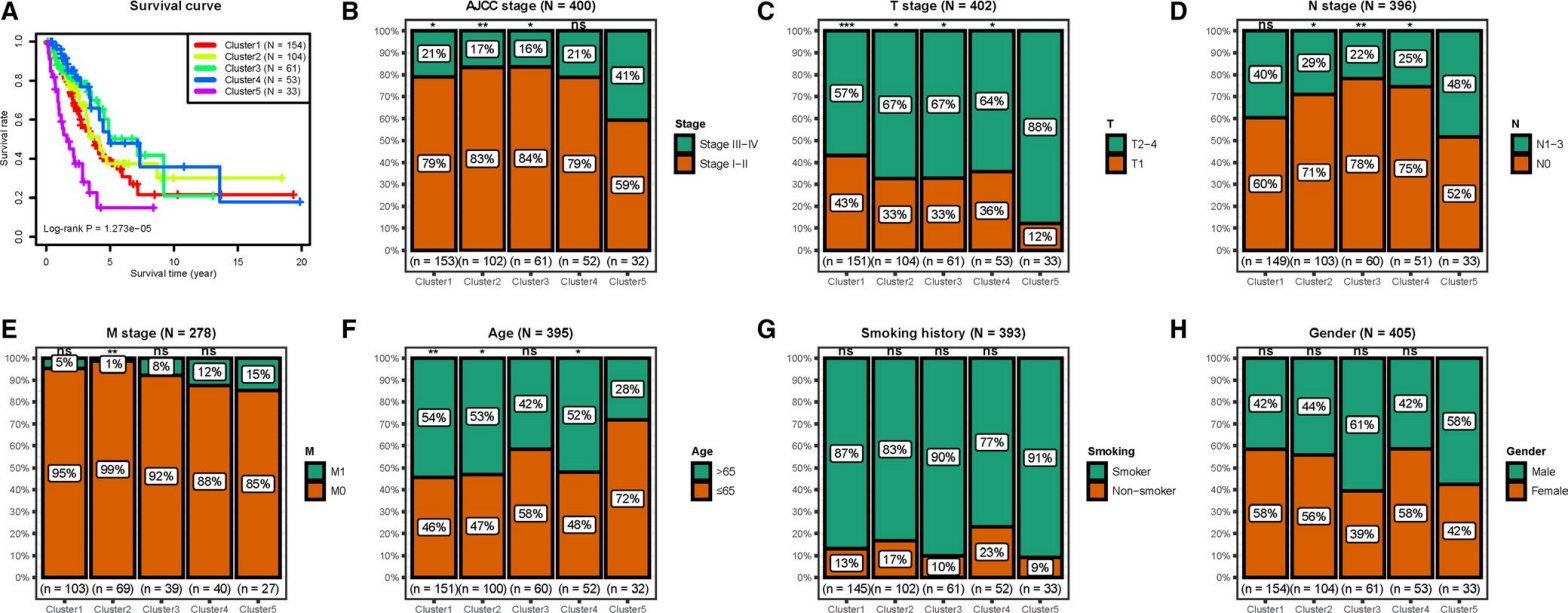
Analyses of the survival and clinical features of the five clusters. A, Survival curves comparing different clusters (log‐rank *P* = 1.273e‐05). B‐H, The distribution proportions of (B) AJCC stage, (C) T stage, (D) N stage, (E) M stage, (F) age, (G) smoking history, and (H) gender in each cluster. Chi‐square test was performed to assess the statistical significance of differences in clinicopathological distribution between patients in cluster 5 and other clusters. ns, not significant; *P < .05; **P < .01; ***P < .001

The Kaplan‐Meier method and log‐rank test demonstrated that the differences in survival among the five clusters were significant (*P* < .001). Figure 4A shows the survival curves, and Figure 4B‐G shows the intracluster proportions of T stage, N stage, M stage, AJCC stage, age, and smoking history in each group. We observed that cluster 5 was associated with the worst clinical outcome despite its relatively younger age distribution; clusters 3 and 4 showed the best clinical outcome, and clusters 1 and 2 showed moderate clinical outcomes. In addition, among the five clusters, cluster 5 contained a significantly lower proportion of patients in the T1 stage and significantly more patients with advanced AJCC and N stage tumors than most of the other clusters. In addition, cluster 5 tended to contain samples of higher M categories, albeit not statistically significant. Detailed results of the Chi‐square test are shown in Table S6. Notably, both survival analysis and the intracluster proportions of clinical parameters indicated that cluster 5 was a group with methylation subtypes predicting poor outcomes.

### 3.4 Identification of cluster‐specific CpG sites

**FIGURE 5:**
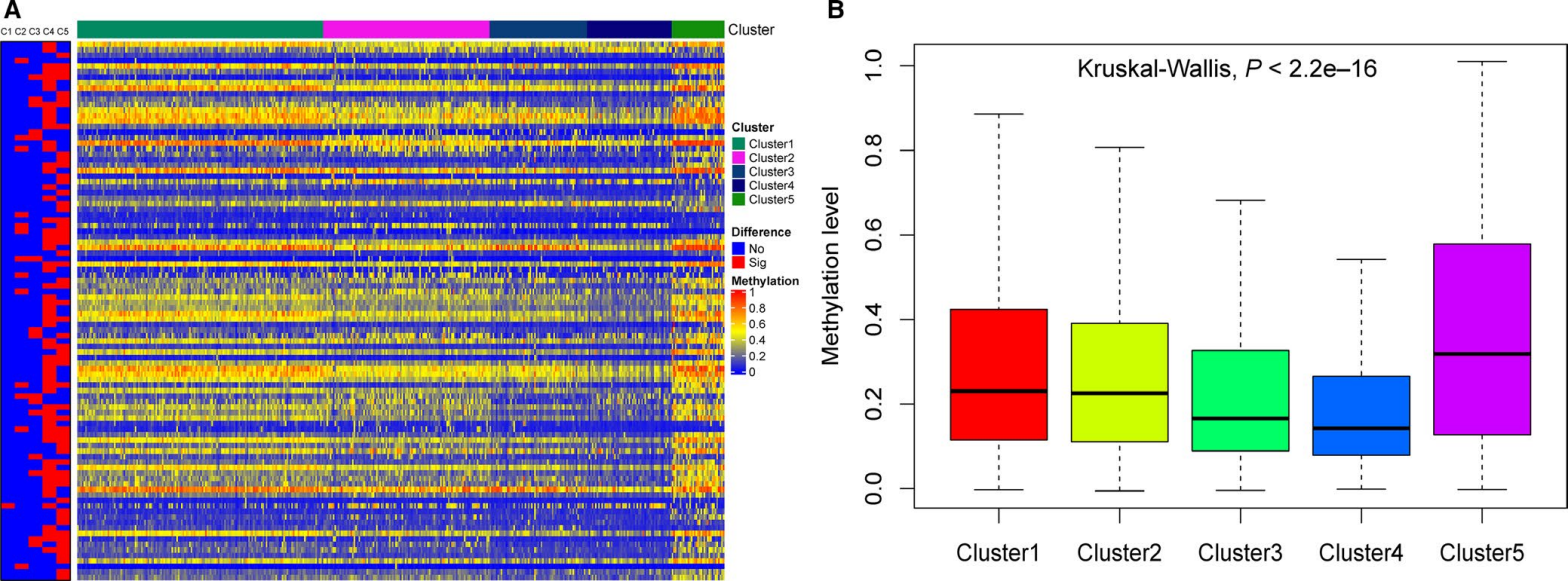
Cluster‐specific CpGs of each cluster. A, Heat map showing the expression of cluster‐specific CpG sites. The red block in the left panel represents the specific CpGs for clusters 1‐5 from left to right. C1, Cluster 1; C2, Cluster 2; C3, Cluster 3; C4, Cluster 4; and C5, Cluster 5. B, Box plot showing that cluster 5 had the highest methylation level among the five groups

After the pairwise comparison, 98 cluster‐specific CpG sites were selected. A heat map based on the methylation status of these 98 CpGs in each cluster is shown in Figure 5A and detailed data are shown in Table S7.

### 3.5 Construction and evaluation of the LUAD risk assessment model

Previous results have demonstrated cluster 5 to be most closely associated with poor clinical outcomes among the five groups. Cluster 5 showed the highest methylation level (*P* < 2.2e–16) among the 98 specific sites (Figure 5B). Hence, we selected the cluster‐specific CpG sites in cluster 5 to establish a prognosis predictive model for patients with LUAD. Next, we performed stepwise model selection and selected 16‐CpGs as the predictive signature. The 16‐CpG sites and multivariate Cox regression data are shown in Table 2 and Table S8. The coefficients of the risk score were estimated using multivariate Cox hazard model analysis and the risk score was calculated as follows: Risk score = 1.588396*cg02829654 + 3.121044*cg03270167 + 2.010610*cg03476195 + 7.476726*cg06352750 − 1.502692*cg06971096 − 2.941728*cg08578023 − 3.934218*cg09450020 − 2.336885* cg12914014 − 9.741084*cg14446712 + 2.686969*cg18328334 − 2.282416*cg19219437 + 2.135792*cg19868691 − 2.367863*cg20938359 + 1.898840*cg24928378 + 1.602154*cg26202340 + 2.998323*cg27217148.

**TABLE 2:** Detailed information of the 16 prognostic CpG sites

ID	Gene symbol	Chromosome	Start	End	Feature type
cg02829654	LYST	chr1	235883639	235883640	—
cg03270167	RAMP1	chr2	237860273	237860274	S_Shore
cg03476195	ANK2	chr4	113049589	113049590	—
cg06352750	SDPR	chr2	191847200	191847201	—
cg06971096	PTPRN	chr2	219308869	219308870	N_Shore
cg08578023	CTSS	chr1	150765697	150765698	—
cg09450020	STEAP2	chr7	90212121	90212122	Island
cg12914014	USP7	chr16	8964508	8964509	S_Shore
cg14446712	SDPR	chr2	191847432	191847433	—
cg18328334	TNS1	chr2	217943929	217943930	—
cg19219437	PCOLCE2	chr3	142888873	142888874	Island
cg19868691	WNT10A	chr2	218879875	218879876	N_Shore
cg20938359	SLC6A12	chr12	213084	213085	—
cg24928378	RND1	chr12	48865632	48865633	—
cg26202340	TIRAP	chr11	126282462	126282463	N_Shore
cg27217148	PCGF6	chr10	103351401	103351402	S_Shore

**FIGURE 6:**
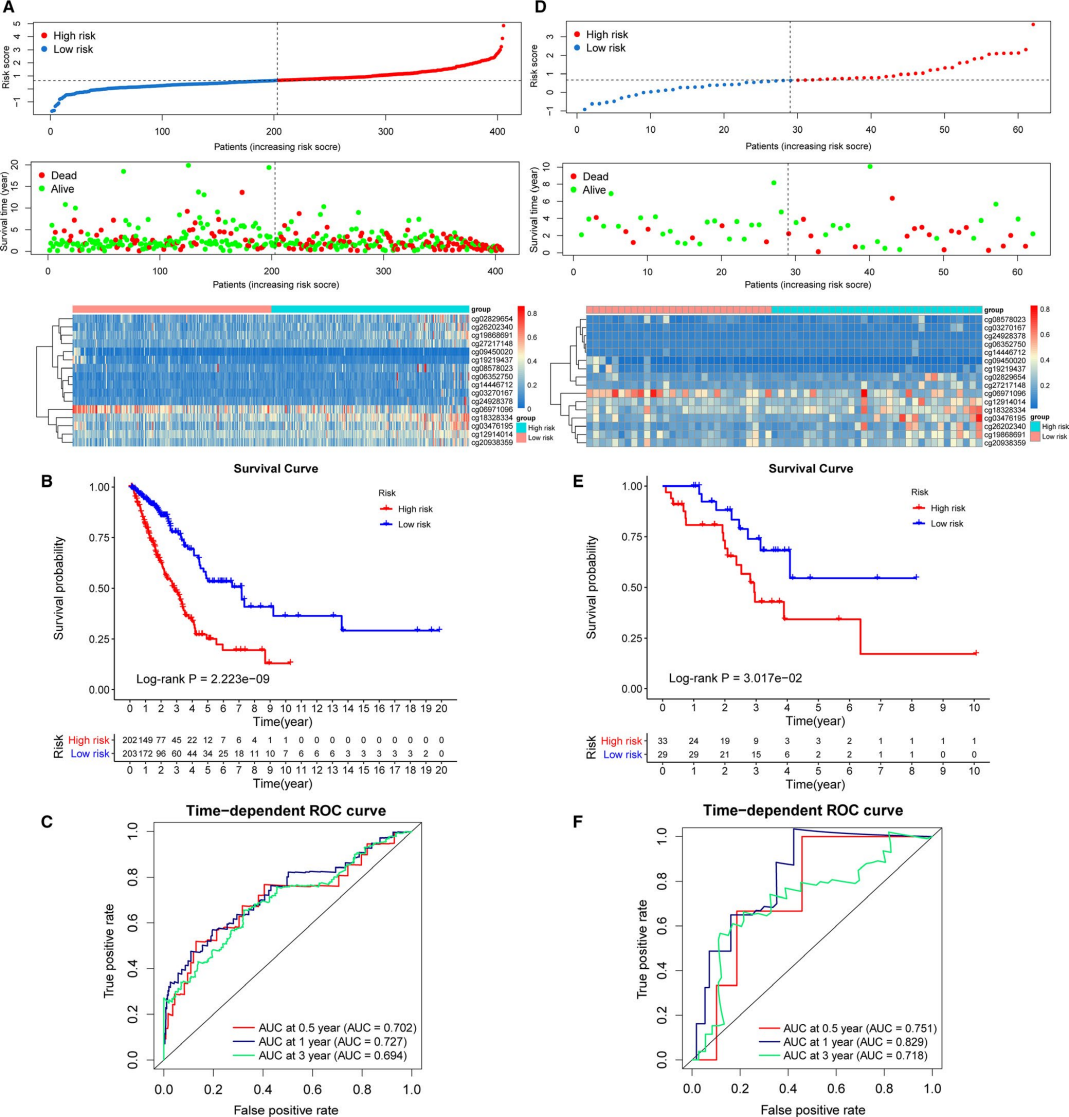
Construction of prognostic prediction model and model validation. A, Risk score (top), overall survival (middle) of patients, and CpG methylation (bottom) in the training set. B, Survival curves showing that the prognostic model clearly distinguished patients in high‐risk and low‐risk groups (log‐rank *P* = 2.223e‐09). C, Time‐dependent ROC curves for patients with LUAD in the training set. The AUC in 0.5, 1, and 3 y were 0.702, 0.727, and 0.694, respectively. D, Risk score (top), overall survival (OS) (middle) of patients, and CpG methylation (bottom) in the test set. E, Survival curves showing that the prognostic model clearly distinguished patients in high‐risk and low‐risk groups (log‐rank *P* = 3.017e‐02). F, Time‐dependent ROC curves for patients with LUAD in the test set. The AUC in 0.5, 1, and 3 y were 0.751, 0.829, and 0.718, respectively

The risk score of each sample was calculated according to the risk score formula. Selecting the median value as the cut‐off, all samples in the training set were divided into high‐risk and low‐risk groups (Figure 6A). As shown in Figure 6A, patients with high‐risk scores showed higher mortality rates than those with low‐risk scores, and the CpGs tended to be hypermethylated in the high‐risk group. The results of the survival analysis (Figure 6B) revealed significant differences between the two groups (*P* < .001). The prognostic performance of the methylation signature and risk scoring models was determined by comparing the area under the respective ROC curves (AUC). The AUC of the prognostic risk assessment model for 0.5, 1, and 3 years were 0.702, 0.727, and 0.694, respectively (Figure 6C).

The prognostic signature was further validated in the test set. The risk scores of 62 patients in the test set were calculated using the risk score formula. Then, the patients were divided into high‐risk and low‐risk groups according to the cut‐off in the training group. Figure 6D shows the risk score distribution, patient survival status, and methylation profile in the test set. The Kaplan‐Meier survival curves showed that patients with LUAD in the high‐risk group had significantly lower survival probability than those in the low‐risk group (*P* < .05) (Figure 6E). In addition, the AUC of the test set for 0.5,1, and 3 years were 0.751, 0.829, and 0.718, respectively, indicating the high sensitivity and specificity of the 16‐CpG methylation signature (Figure 6F).

### 3.6 Stratified survival analysis

**FIGURE 7:**
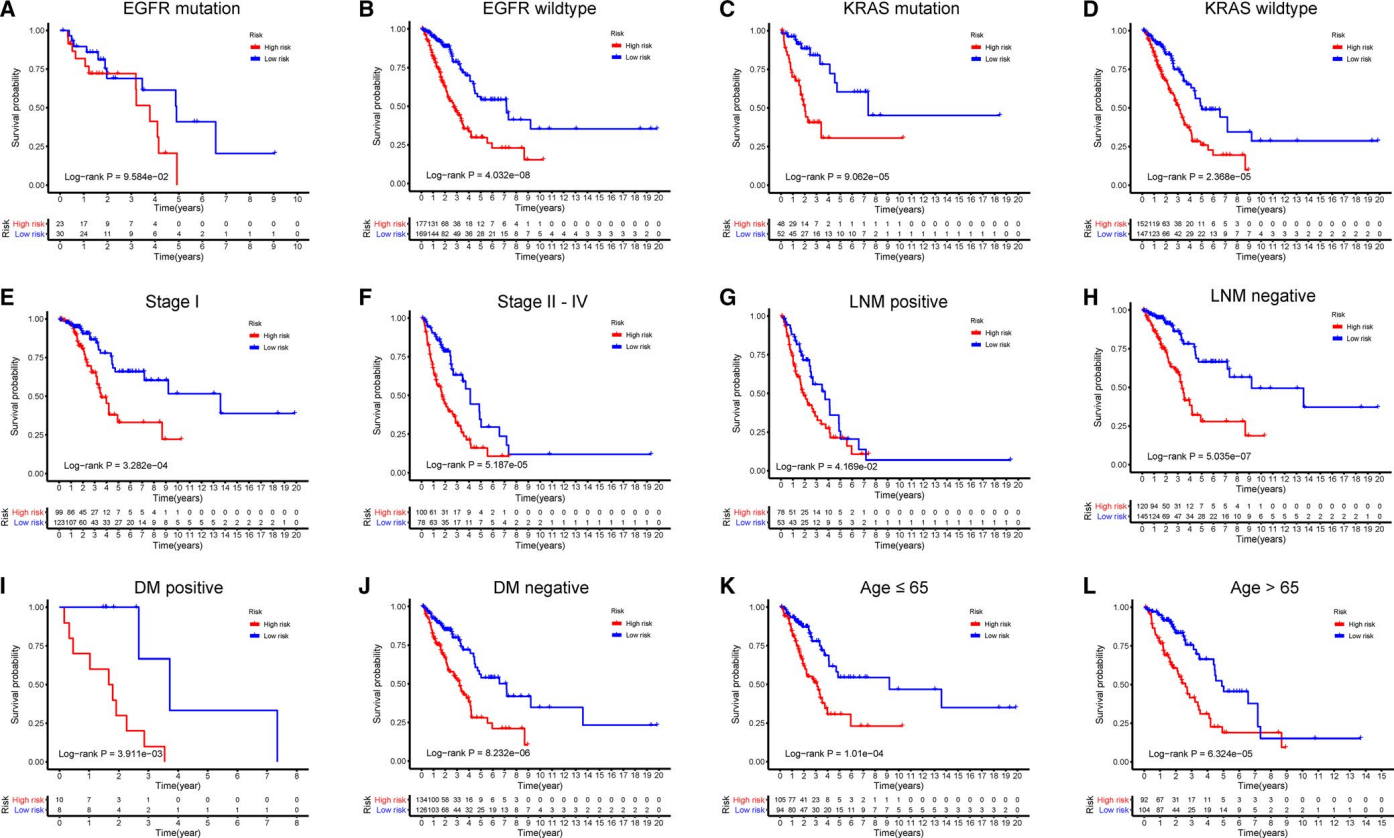
Stratified analysis for patients with LUAD in the training set. Patients were assigned to different subgroups according to clinicopathological risk factors. A‐B, *EGFR* mutation status. C‐D, *KRAS* mutation status. E‐F, AJCC stage I and stage II‐IV. G‐H, Lymph node metastatic (LNM)‐positive and ‐negative. I‐J, Distant metastatic (DM)‐positive and ‐negative. K‐L, Age ≤ 65 and > 65 y

To evaluate the predictive performance of the risk score model more precisely, we performed a stratified survival analysis. Patients were divided into different subgroups based on their clinical and pathological parameters, including *EGFR* status, *KRAS* status, AJCC stage, LNM condition, DM condition, and diagnostic age. The mutation data of *EGFR* and *KRAS* are shown in Table S9 and the survival curves regarding *EGFR* and *KRAS* mutation status are shown in Figure 7A–D, in which the high‐risk group correlated with unfavorable survival in the *EGFR* wild‐type, *KRAS* mutation, and *KRAS* wild‐type subgroups (*P* < .001). The high‐risk group tended to have worse OS than the low‐risk group in the *EGFR* mutation subgroup, although the result was not statistically significant (*P* > .05), which may be because of the relatively small subgroup size (Figure 7A). Patients in the high‐risk group generally showed significantly worse survival than the low‐risk group in subgroup stage I and stage II‐IV (*P* < .001) (Figure 7E,F). For patients in LNM‐positive or LNM‐negative subgroup, OS was significantly longer for patients in the low‐risk group than in the high‐risk group (*P* < .05) (Figure 7G,H). Similarly, patients in the low‐risk group generally showed better clinical outcomes than those in the high‐risk group in both the DM‐positive and DM‐negative subgroups and age > 65 and ≤65 years subgroups (*P* < .01) (Figure 7I‐L). All the above results indicated the stable predictive capacity of the risk score model.

### 3.7 Relationships between the risk score model and important risk factors

**FIGURE 8:**
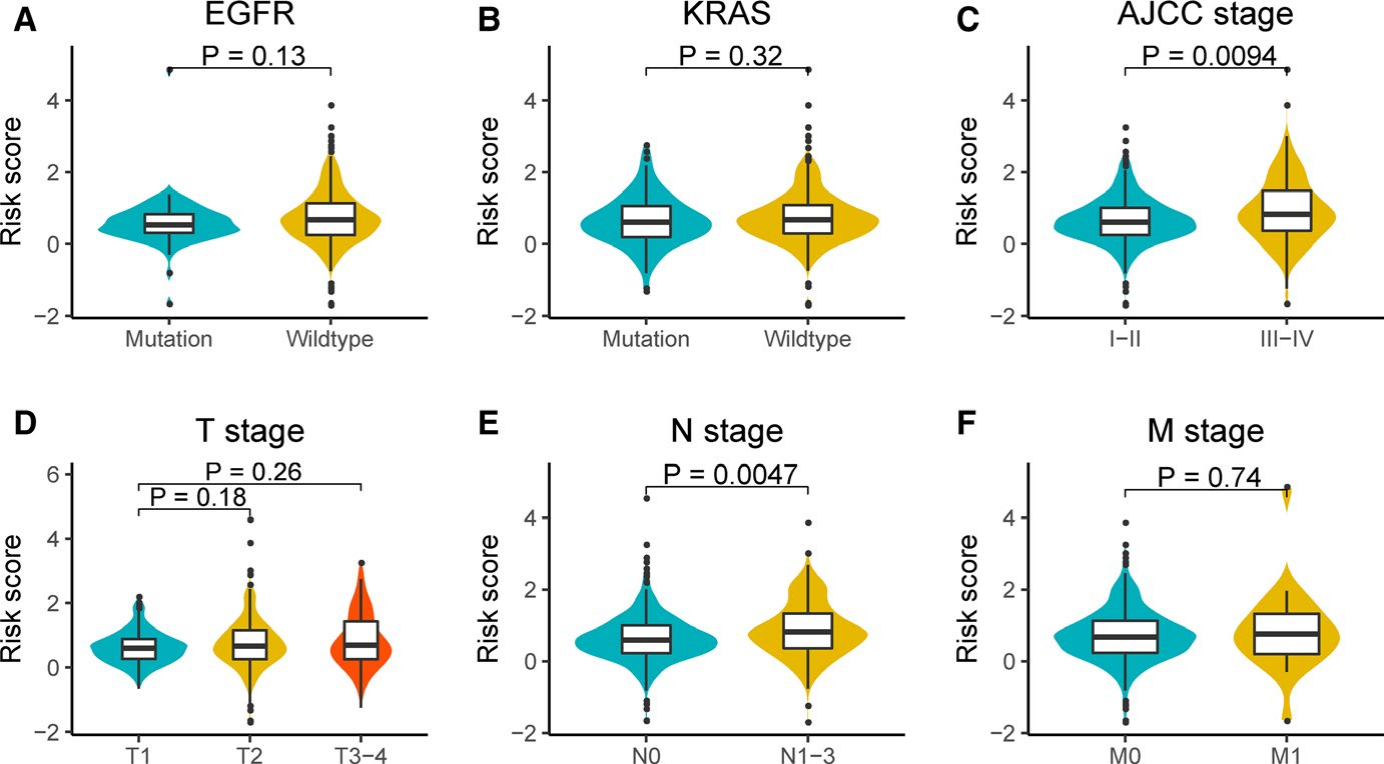
Relationships between the risk score model and important risk factors. A, *EGFR* mutation status. B, *KRAS* mutation status. C, AJCC stage. D, T stage. E, N stage. F, M stage. The differences were compared using the Wilcoxon test

Further investigation showed correlations between the risk score model and the AJCC stage (*P* < .01), as well as the N stage (*P* < .01) (Figure 8C,E). The distributions of risk score between different groups, including *EGFR* mutation, *KRAS* mutation, T stage, and M stage, are shown in Figure 8A,B,D,F.

### 3.8 Prognostic nomogram for OS

**FIGURE 9:**
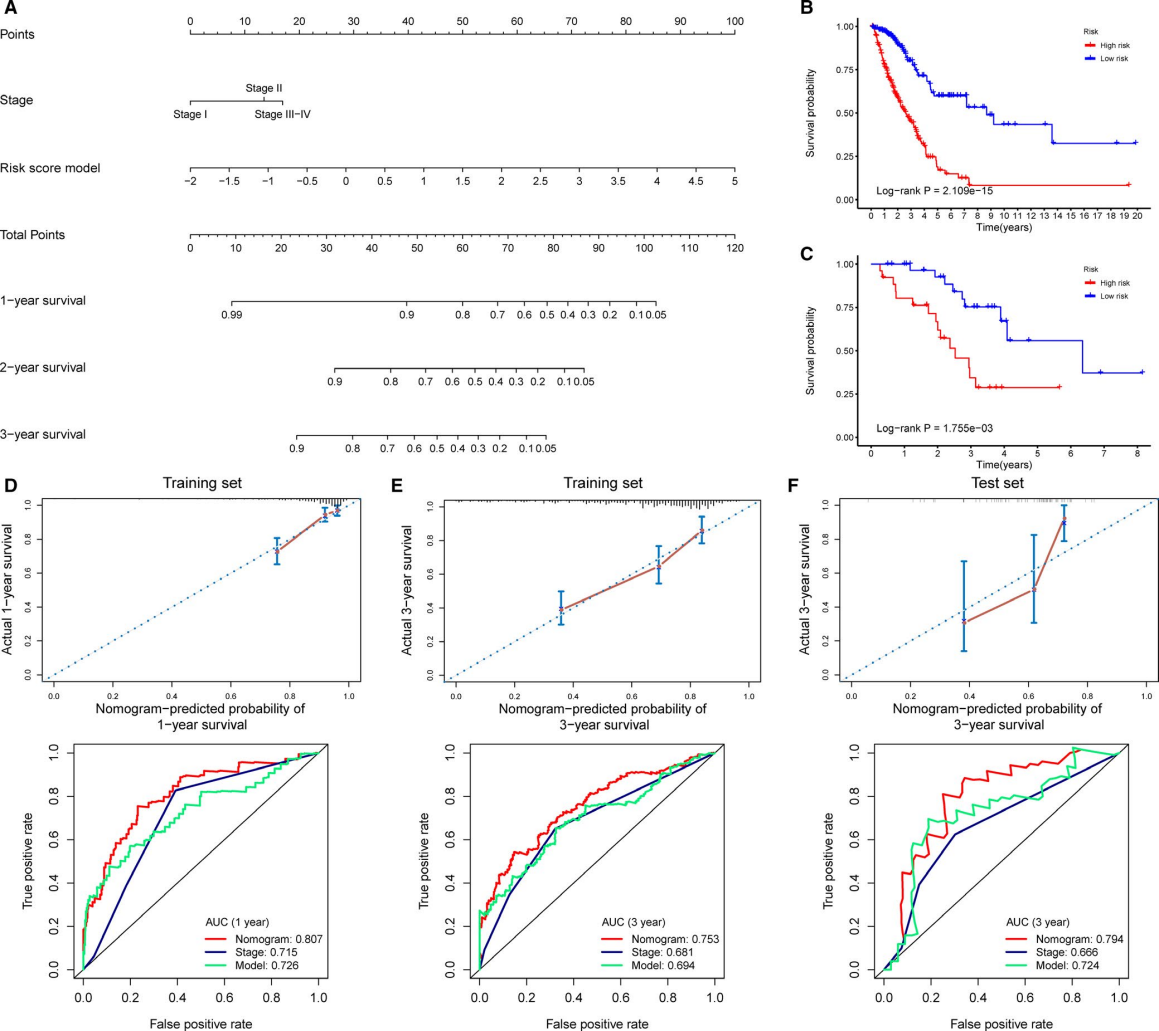
Nomogram development and evaluation. A, A nomogram to predict OS of patients with LUAD. B‐C, Kaplan‐Meier survival analysis for patients in the training set and the test set according to the risk scores calculated from the nomogram. D‐E, Calibration curves and time‐dependent ROC curves at 1 and 3 y for patients in the training set. F, Calibration curves and time‐dependent ROC curves at 3 y for patients in the test set. ROC plots in D‐F compare AUC values of the nomogram, AJCC stage, and the risk score model. The AUC values of the risk score model and the *X*‐axis range in C differ slightly from those in Figure 6 because of the removal of samples with missing AJCC stage data

Using the data of the training set, we developed a prognostic nomogram for OS. The nomogram was constructed using two parameters: AJCC stage and the risk score model (Figure 9A). The total points of the nomogram were calculated from the sum of the points of individual variables generated based on multivariate analysis. Using the median risk score from the nomogram as the cut‐off, patients with LUAD in the training and test sets were divided into the high‐risk and low‐risk groups, respectively. Kaplan‐Meier survival analysis showed worse OS in the high‐risk group than in the low‐risk group in both sets (Figure 9B,C). The C‐index was 0.744 for the training set (95% confidence interval [CI] = 0.701‐0.788) and 0.762 for the test set (95% CI = 0.666‐0.859). The calibration plots for the predicted probability of 1‐year or 3‐year survival in the training set showed optimal overlap with the actual observation (Figure 9D‐E). The calibration curve for the probability of survival for 3 years in the test set also showed good agreement between prediction and observation (Figure 9F). In addition, the time‐dependent ROC curves in both training (1 or 3 years) and test sets (3 years) indicated that the discriminatory ability of the nomogram was superior to that of the 16‐CpG‐based model or AJCC stage at these time points. These results indicated the robust discriminatory ability and calibration of the nomogram.

### 3.9 GSEA and construction of the pathway network

**FIGURE 10:**
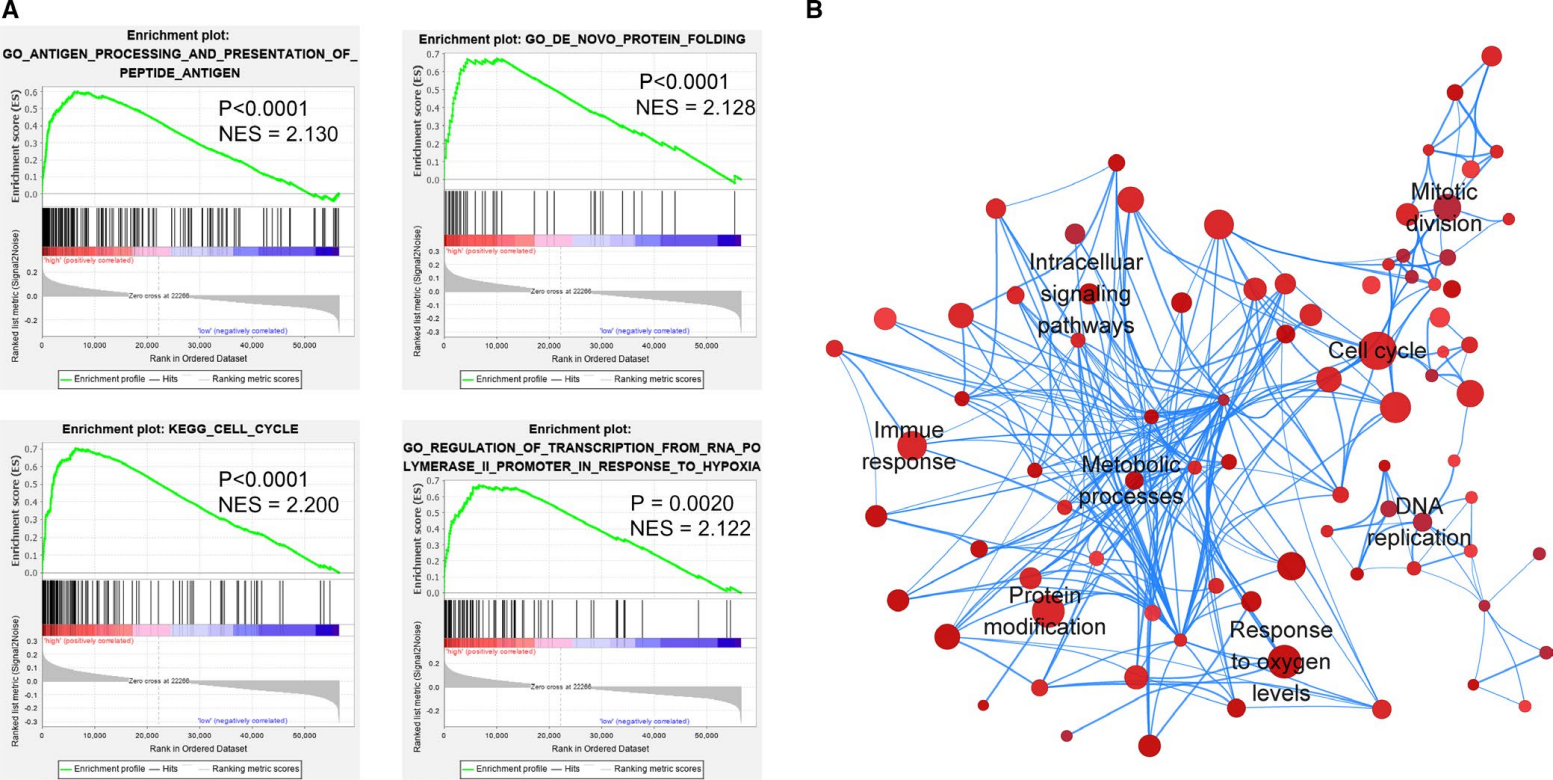
DNA methylation signature‐associated pathways in high‐risk LUAD. A, Representative pathways enriched in the high‐risk LUAD samples using GSEA. NES, normalized enrichment score. B, Network diagram showing the overall pathway interactions in the high‐risk group according to the GSEA results. Nodes represent the pathways with *P* < .05 and *FDR* < 0.1. Node size represents the gene number enriched in the corresponding pathway. Color depth represents the *P* value of the selected gene set. The deeper the color, smaller is the *P* value. The edge thickness represents the gene number shared by both pathways

Considering that the 16‐CpG signature‐derived risk score system may be closely related to multiple important pathways, we conducted a GSEA between the high‐risk group and low‐risk group to identify the important signaling pathways associated with these CpG sites. As shown in Figure 10A, multiple pathways, such as cell cycle, antigen processing and presentation, protein folding, and ketone metabolic process, were enriched in LUAD patients with high‐risk score. A pathway network revealing the overall interaction of these representative pathways is shown in Figure 10B. The detailed GSEA results of the 396 available LUAD samples are shown in Table S10.

## 4 DISCUSSION

The present study demonstrates the potential of a methylation signature in determining the diagnosis, prognosis, and treatment of LUAD. DNA hypermethylation is a hallmark of tumorigenesis and may contribute to early diagnosis and prognosis of LUAD.^27^ Furthermore, the reversibility of DNA methylation is expected to be the breakthrough point for targeted therapy.^28^ Several methylation biomarkers for LUAD, such as *RASSF1A, MGMT*, and *CDKN2A*, have been shown to be associated with the prognosis of LUAD.^29^, ^30^, ^31^ However, owing to the complexity and heterogeneity of LUAD, the prognosis should be determined using a CpG biomarker panel rather than a single CpG biomarker.

In total, we obtained 16 prognosis prediction CpG sites and validated their predictive capability in the training and test cohorts. First, we identified the independent prognostic CpG sites using univariate and multivariate Cox regression models. Next, we performed consensus clustering to identify LUAD subgroups based on the most variable CpGs. Subsequently, we analyzed the survival situation and clinical parameters of each group. All the results suggested that cluster 5 had the lowest survival probability, the most advanced TNM stage, and the highest methylation level. Therefore, we selected cluster 5 for further investigations and detected 56 cluster‐specific CpG sites for subsequent stepwise regression and model construction. The consensus clustering method applied in this study is a reliable unsupervised resampling‐based classification methodology, which successfully categorized the LUAD population into nonoverlapping subgroups with distinct methylation profiles.

After model construction, we performed Kaplan‐Meier survival test, stratified survival analysis, and ROC analysis in the training set, the results of which were indicative of considerable prognostic ability. The Wilcoxon test also showed that the risk score model may be related to AJCC stage and N stage. Furthermore, we also validated our risk score model in the test set, and AUC values at 0.5, 1, and 3 years were 0.751, 0.829, and 0.718, respectively, indicating the prognostic value of the DNA methylation signature in LUAD.

The high heterogeneity of LUAD at the population level represents a limitation for precisely predicting OS for LUAD. Although the performance of the risk score model was satisfactory, generally a prognostic model with ROC curve AUC < 0.75 is not clinically useful.^32^ Thus, a nomogram that can integrate the 16‐CpG‐based model and AJCC stage of patients with LUAD was developed to circumvent this problem. Further analysis showed that the AUC values of the nomogram model were > 0.75 in both the training (1 or 3 years) and test sets (3 years), which revealed the feasibility of using the nomogram model for clinical applications.

As shown in Table 2, 15 genes corresponded to the 16‐CpG signature (cg06352750 and cg14446712 are both on the promoter region of *SDPR*). The PubMed was searched for articles on these 15 genes related to LUAD. Up till 25 February 2020, with the exception of *RAMP1, SDPR, USP7, TNS1*, and *PCGF6*, the other 10 genes were identified for the first time as LUAD‐related genes. In addition, other than *SDPR* and *USP7*, methylation of the 13 other genes was implicated for the first time to be related to LUAD prognosis. Two CpG sites of *SDPR* were included in the prognostic model, as *SDPR,* a gene with frequent methylation, may be closely associated with patient survival. A previous study has shown SDPR to be a caveolar protein that causes deformation of caveolae and extensive tubulation of the plasma membrane.^33^ Li et al reported that SDPR was induced by Fhl1, and that SDPR expression was suppressed in tumors of the breast, kidney, and prostate.^34^ Wang et al identified SDPR as an independent survival prognostic factor, the expression of which may be regulated by GATA‐binding protein 2.^35^

Regulated by the m6A demethylase FTO, *USP7* is an oncogene that promotes cancer cell proliferation by deubiquitinating Ki‐67.^36^, ^37^ Hong et al observed that the CpG site cg17183999 of *USP7* was associated with *EGFR* mutation in patients with LUAD.^38^ Previous studies have demonstrated that RAMP1 affects glycosylation and transfer of calcitonin‐like receptor (CLR) to the cell surface.^39^, ^40^ TNS1 overexpression has been found to contribute to the invasion and metastasis of LUAD cells.^41^ PCGF6, a member of the Polycomb group (PcG) family of transcriptional repressors, has been identified in the Polycomb repressive complexes (PRC1) that possesses H3K9 methyltransferase and H3K4 demethylase activities. PCGF6 represses dendritic cell activation and positively regulates cell quiescence.^42^ Llabata et al identified the tumor‐suppressive effect of PCGF6 on the MYC pathway in LUAD.^43^

Compared to a previous study,^9^ the 16‐CpG methylation signature in this study was validated using an external test dataset. In addition, a targeted therapy actually benefitted a patient population with specific molecular characteristics. For example, despite tyrosine kinase inhibitors (TKIs) targeting EGFR can effectively extend the survival time and improve the life quality of NSCLC patients; only about 10%‐20% patients with non‐EGFR mutation benefitted from it.^44^, ^45^ Hence, instead of simply selecting a series of prognosis‐related CpGs and establishing a predictive model using mathematical modeling,^8^, ^9^ we comprehensively analyzed the methylation profile, survival data, and clinical features of different subpopulations and characterized their specific methylation characteristics. This is more meaningful for personalized molecular treatment of patients with LUAD.

The mechanisms that contribute to different survival rates between two stratified groups were also investigated. The GSEA results indicated that some important pathways and biological processes were significantly enriched in the high‐risk group, including cell cycle, protein folding, antigen presentation, metabolic pathway, Wnt signaling pathway, and NF‐κB signaling pathway. Some of these significantly enriched pathways and biological processes possibly involve the functions of several of the abovementioned genes, such as *USP7, RAMP1*, and *PCGF6*. Furthermore, the pathway network constructed in this study may inspire future studies on molecular mechanisms and development of precise therapies for LUAD.

Although the predictive performance of the DNA methylation signature was encouraging, our study has certain limitations. First, only the CpGs overlapping between the 450 K array and 27 K array were retained for experimental analysis, and many data were removed due to poor data integrity. Therefore, our candidate CpG sites are only a proportion of the entire CpG sites in the genome of patients with LUAD. Second, the biological mechanism of action of these biomarkers, as well as the role of the associated pathways, is still unknown. Third, further validation, such as prospective studies and clinical trials in different LUAD populations, is still required.

In summary, we systematically analyzed the LUAD methylation data from TCGA, identified a 16‐CpG prognostic signature, and established a prognostic risk model. The novel molecular landscape identified in this study and the newly developed risk model may be used as tools for assessing the prognosis of patients with LUAD. Furthermore, our observations may act as important bioinformatics basis for further investigations regarding molecular therapeutics.

## CONFLICT OF INTEREST

The authors have no conflict of interest to declare.

## AUTHOR CONTRIBUTIONS

XW conceived and designed the work. QDC and ZYZ collected and analyzed the data. XP and YQZ prepared all the tables and figures. QDC, BXH, HX, and PFZ wrote the manuscript. All authors read and approved the final manuscript.

## Supporting information

Table S1

Table S2

Table S3

Table S4

Table S5

Table S6

Table S7

Table S8

Table S9

Table S10

## Data Availability

The data that support the findings of this study are openly available in TCGA and UCSC Xena databases.
